# Tumor Innervation: From Bystander to Emerging Therapeutic Target for Cancer

**DOI:** 10.3390/ijms26189257

**Published:** 2025-09-22

**Authors:** Zoey Zeyuan Ji, Max Kam-Kwan Chan, Philip Chiu-Tsun Tang, Calvin Sze-Hang Ng, Chunjie Li, Dongmei Zhang, David J. Nikolic-Paterson, Ka-Fai To, Xiaohua Jiang, Patrick Ming-Kuen Tang

**Affiliations:** 1 Department of Anatomical and Cellular Pathology, State Key Laboratory of Translational Oncology, The Chinese University of Hong Kong, Hong Kong; zoeyji@link.cuhk.edu.hk (Z.Z.J.); 1155149761@link.cuhk.edu.hk (M.K.-K.C.); philtang@link.cuhk.edu.hk (P.C.-T.T.); kfto@cuhk.edu.hk (K.-F.T.); 2 Department of Surgery, The Chinese University of Hong Kong, Hong Kong; calvinng@surgery.cuhk.edu.hk; 3 Department of Head and Neck Oncology, West China Hospital of Stomatology, Sichuan University, Chengdu 610065, China; lichunjie@scu.edu.cn; 4 College of Pharmacy, Jinan University, Guangzhou 510632, China; dmzhang701@jnu.edu.cn; 5 Department of Nephrology and Monash University Department of Medicine, Monash Medical Centre, Clayton, VIC 3168, Australia; david.nikolic-paterson@monash.edu; 6 Key Laboratory for Regenerative Medicine of the Ministry of Education of China, School of Biomedical Sciences, Faculty of Medicine, The Chinese University of Hong Kong, Hong Kong; xjiang@cuhk.edu.hk; 7 Li Ka Shing Institute of Health Sciences, The Chinese University of Hong Kong, Shatin, Hong Kong; 8 Peter Hung Pain Research Institute, The Chinese University of Hong Kong, Hong Kong

**Keywords:** tumor innervation, tumor–nerve crosstalk, denervation therapy

## Abstract

Innervation is ubiquitous in diseased tissues, including cancer. Increasing evidence suggests that innervation not only plays a direct role in cancer pain, but is also closely related to disease progression, including cancer growth, metastasis, and drug resistance. At the molecular level, tumor-associated nerves can interact with cancer cells and the tumor microenvironment through neurotrophic factors, thereby promoting tumor occurrence and development, and represent a potential intervention for solid tumors with nerve enrichment. By dissecting the transcriptome dynamics of cancer-associated neurons with single cell resolution, numbers of novel therapeutic targets for tumor denervation have been uncovered, including a novel phenomenon—Macrophage to Neuron-like cell Transition (MNT). This review systematically summarizes the latest research findings of tumor denervation, from molecular mechanisms to the innovative denervation strategies, paving the way for novel, safe, and effective cancer treatments in the clinic.

## 1. Introduction

Cancer remains a leading cause of morbidity and mortality worldwide [1]. Solid tumors develop from genetic mutations that enable primary cancer cells to acquire proliferative and metastatic capacities [2]. However, cancer progression is far more complex than the proliferation of malignant cells. Increasingly, the tumor microenvironment (TME) is recognized as a critical player in promoting tumor growth, immune evasion, and metastasis [3,4,5]. Among its many components, tumor innervation—the interaction between nerves and the tumor—has emerged as a key modulator of cancer progression [6,7,8].

Tumor innervation encompasses both perineural invasion (PNI) [9,10], where cancer cells invade pre-existing nerves, and spontaneous nerve formation (SNF) [11,12]. SNF refers to the de novo formation of nerve fibers within tumors, independent of pre-existing neural structures [12,13]. Unlike neoneurogenesis, which involves the tumor-induced ingrowth of external nerves via neurotrophic signaling [14]. It also differs from nerve infiltration, which denotes the passive extension of pre-existing nerves into tumor tissue without de novo fiber formation [15]. PNI is a hallmark of aggressive cancers like pancreatic [16,17], prostate [18,19], and colorectal cancer [19], where it correlates with poor prognosis and recurrence. Meanwhile, nerve formation often results from the recruitment of peripheral nerves or the reprogramming of stromal cells into neuron-like phenotypes. These processes enable cancer-associated nerves to promote tumor progression, including tumor growth [20,21], invasion [8], metastasis [22], and stimulation of angiogenesis [23].

Clinically, nerve density in tumor has been linked to worse outcomes [24,25,26], including higher rates of metastasis, recurrence, and mortality. This is particularly evident in highly innervated cancers such as pancreatic cancer [27], where nearly all tumors exhibit significant neural infiltration, as well as in head and neck [24], prostate [28,29], and colorectal cancers [30]. Cancer-associated nerves influence the TME through bidirectional signaling [31]. Nerves not only respond to cancer-driven neurogenesis but also secrete neurotransmitters and growth factors that further promote tumor growth, survival, and immune suppression (Table 1).

The intricate crosstalk between nerves and the TME presents unique opportunities for therapeutic intervention. Emerging strategies aim to disrupt nerve–tumor interactions by targeting neurotrophic signaling pathways, axon guidance molecules, and nerve infiltration mechanisms. In this review, we summarize the biological mechanisms underpinning tumor innervation, highlight its clinical significance, and explore therapeutic approaches that capitalize on these insights. Understanding the role of nerves in cancer progression will pave the way for innovative treatments and improve patient outcomes.

## 2. Tumor Innervation

### 2.1. Tumor-Associated Nerves

Nerves in tumors represent an emerging hallmark of cancer progression, with increasing recognition of their role in shaping the TME [44]. Tumor innervation refers to the presence of nerves either infiltrating from surrounding tissues or forming new neurons within the tumor. These nerves include sympathetic, parasympathetic, and sensory fibers that actively interact with tumor cells and stromal components [9].

**Figure 1:**
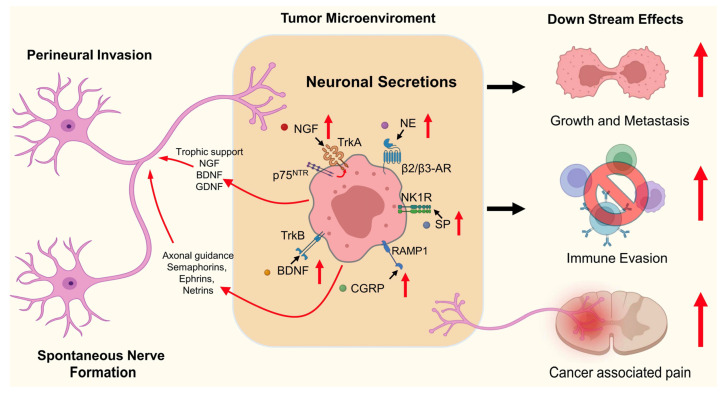
The pathogenic roles and molecular mechanisms of tumor-associated nerves in cancer. Tumor-associated nerves actively secrete neuronal transmitters into the tumor microenvironment, including calcitonin gene-related peptide (CGRP), nerve growth factor (NGF), brain-derived neurotrophic factor (BDNF), norepinephrine (NE), and substance P (SP). They can directly activates their respective receptors on the cancer cells (e.g., TrkA (NGF), TrkB (BDNF), β2/β3-adrenergic receptors (NE), NK1R (SP), and the RAMP1/CLR complex (CGRP)) for promoting tumor growth, metastasis, immune evasion, as well as cancer pain formation. Neurotrophic factors (NGF, BDNF, GDNF) also provide trophic support for nerve survival and growth, whereas axon guidance molecules (e.g., semaphorins, ephrins, netrins) regulate directional neural ingrowth within tumors. Red upward arrows: Upregulation of neuronal secretions and their effects. Curved red arrows: Reciprocal support for nerve growth and ingrowth. Straight black arrows: Direction of signaling from neuron secretions to receptors. Black arrows (to right side): Contribution of neuronal sig-naling to growth/metastasis, immune evasion, and cancer pain.

Two primary forms of tumor innervation are described (Figure 1). PNI is a well-characterized process in which cancer cells invade and migrate along existing nerves, often seen in aggressive cancers such as pancreatic, prostate, and head and neck cancers [18]. It is associated with poor prognosis, increased recurrence rates, and distant metastasis. In contrast, SNF refers to the growth of nerve fibers within the tumor, driven by cancer-secreted neurotrophic factors or reprogramming of non-neuronal cells like macrophages [12] and cancer stem cells [45].

The density of tumor-associated nerves correlates with disease severity and patient outcomes [46,47]. In cancers such as pancreatic and colorectal tumors, a high degree of innervation is associated with enhanced tumor growth, immune suppression, and metastatic potential. Understanding these mechanisms provides novel avenues for therapeutic intervention targeting tumor–nerve interactions.

### 2.2. Origins of Tumor-Associated Neurons

#### 2.2.1. PNI

PNI is a distinct pathological phenomenon characterized by the infiltration, encasement, and migration of cancer cells along existing nerve fibers [48]. It is particularly common in malignancies with aggressive clinical behavior, including pancreatic, prostate, colorectal, gastric, breast, and head and neck cancers [35,40,49,50]. Clinically, the presence of PNI strongly correlates with increased tumor recurrence, distant metastasis, therapeutic resistance, and significantly reduced patient survival rates [37,38,40]. Histopathological studies have demonstrated that nearly 100% of pancreatic adenocarcinomas display varying degrees of perineural infiltration [33], a feature strongly associated with debilitating clinical symptoms, especially severe neuropathic pain, impaired organ function, and surgical resection challenges due to intricate nerve entanglement [34]. Similarly, prostate and head-and-neck cancers with pronounced PNI exhibit significantly elevated risk profiles, aggressive disease progression, and reduced overall survival. PNI is orchestrated through complex reciprocal interactions between cancer cells and the neural microenvironment.

Cancer cells actively produce and secrete neurotrophic factors, notably nerve growth factor (NGF), brain-derived neurotrophic factor (BDNF), neurotrophin-3 (NT-3), and glial cell line-derived neurotrophic factor (GDNF). These neurotrophic factors bind to specific high-affinity receptors expressed on neuronal fibers, such as tropomyosin receptor kinase A (TrkA) for NGF and tropomyosin receptor kinase B (TrkB) for BDNF, subsequently promoting nerve sprouting, elongation, and providing chemotactic cues that guide tumor cell migration along neural pathways [16,39,51,52].

Simultaneously, axon guidance molecules, including Semaphorins, Netrins, Ephrins, and SLIT2, known as directing axon pathfinding, facilitate cancer progression [53,54]. For instance, semaphorin 3A and semaphorin 4D have been extensively documented to promote tumor progression [55,56,57], immune suppression [41], and nerve–tumor interactions, contributing directly to PNI progression [58].

Beyond structural invasion, infiltrated nerves actively enhance tumor malignancy by releasing neurotransmitters and neuropeptides, such as NE, acetylcholine, SP, CGRP, and vasoactive intestinal peptide (VIP) [59]. These secreted factors activate specific oncogenic signaling pathways, driving tumor cell proliferation, angiogenesis, immune evasion, and epithelial-to-mesenchymal transition (EMT), thereby promoting metastatic dissemination [60]. Therapeutically, disrupting the molecular interactions underlying PNI has become an attractive strategy. Targeting neurotrophic factor pathways (e.g., NGF/TrkA inhibitors, anti-BDNF monoclonal antibodies), as well as modulating axon guidance signaling (e.g., semaphorin receptor antagonists), has demonstrated potential in preclinical models to attenuate perineural tumor growth, mitigate associated neuropathic pain, and improve responsiveness to conventional therapies [61,62,63,64].

#### 2.2.2. SNF

SNF, distinct from PNI, refers to the de novo generation of nerve fibers within tumors, independent of pre-existing neural structures. This phenomenon emerges intrinsically within the TME, driven by tumor-derived neurotrophic factors such as NGF, GDNF, and vascular endothelial growth factor (VEGF), which stimulate axonal sprouting and nerve fiber formation directly within tumors [13,65,66,67,68,69].

Cancer stem cells (CSCs) exhibit intrinsic neural plasticity, significantly contributing to spontaneous tumor innervation. CSCs are characterized by their remarkable capability to differentiate into multiple cell lineages, including neuron-like phenotypes [45,70,71]. During this neural differentiation process, CSCs express an array of neuronal differentiation markers, such as TUBB3, doublecortin (DCX), microtubule-associated protein 2 (MAP2), nestin, NeuN, and neuronal transcription factors like SOX2 and PAX6 [72,73]. These neuronally differentiated CSCs can structurally integrate into the spontaneously formed intratumoral nerve networks, enhancing the complexity and functionality of the tumor innervation [74,75].

**Figure 2:**
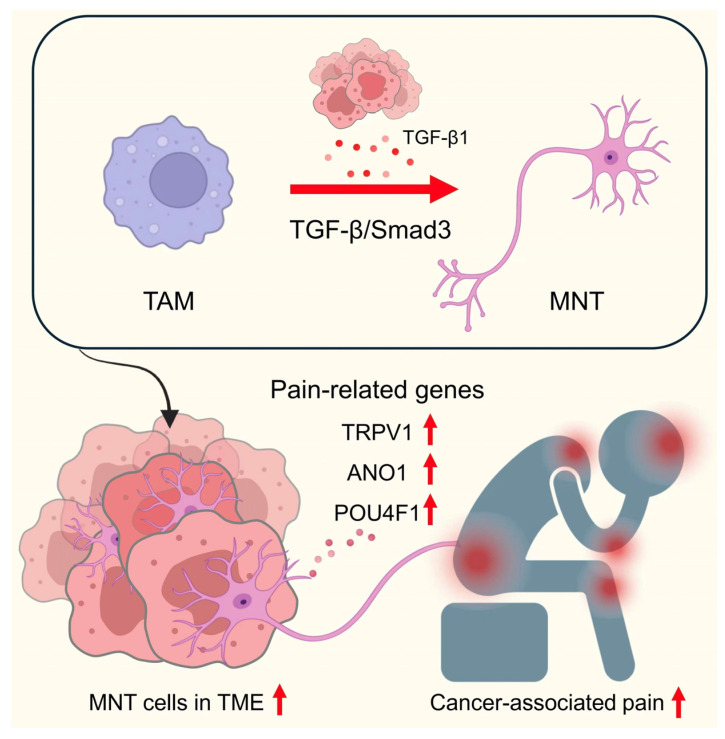
Macrophage to Neuron-like cell Transition (MNT). The tumor-associated macrophages can be further differentiated into functional neuron-like cells in tumor under chronic inflammation via a TGF-β/Smad3 dependent mechanism named MNT, which is primarily induced by tumor cell–secreted TGF-β1. These MNT-derived neurons highly express pain-related genes, including TRPV1, ANO1, POU4F1, and directly contribute to the formation of cancer pain, representing a novel neuro-immune axis in the tumor microenvironment. Straight red arrows: Upregulation of TGF-β signaling. Red upward arrows: Upregulation of gene expression and cancer-associated pain. Black arrows: Direction of cellular transitions and signaling events.

A critical mechanism underpinning SNF involves MNT, wherein tumor-associated macrophages (TAMs) transdifferentiate into neuron-like cells. Direct lineage-tracing studies using LysM-Cre/tdTomato mice confirmed the myeloid origin of these neuron-like cells, excluding contributions from pre-existing neurons or neural progenitors. Single-cell RNA sequencing and pseudotime trajectory analysis further revealed a progressive transcriptional shift from macrophage identity (e.g., CD68, F4/80) toward a neuronal program (TUBB3, DCX, MAP1B, POU4F1), corroborating the transdifferentiation process. Functional assays demonstrated calcium influx in response to nociceptive agonists such as capsaicin (TRPV1) and GSK1016790A (TRPV4), providing physiological evidence of neuronal activity. This remarkable cellular plasticity entails loss of canonical macrophage identity markers (e.g., CD68) and acquisition of neuronal markers including β3-tubulin (TUBB3), doublecortin (DCX), MAP1B, SHANK, and the neuronal transcription factor POU4F1. Initially characterized in lung cancer models using single-cell RNA sequencing and lineage tracing, these MNT cells exhibit functional neuronal characteristics such as nociceptive responses, evidenced by calcium influx upon stimulation with pain-related agonists like capsaicin and TRPV4 activators. Mechanistically, the development of MNT cells is predominantly regulated by the TGF-β1/Smad3 signaling pathway, and genetic ablation of Smad3 in macrophages markedly suppresses MNT formation and reduces tumor-associated pain behaviors [12] (Figure 2).

Parallel evidence from nasopharyngeal carcinoma (NPC) further supports the existence and functional relevance of MNT cells, identifying a neuron-like TAM population regulated through the APOE–TREM2 signaling axis. In this context, tumor-derived apolipoprotein E (APOE) binds to TREM2 on TAMs, triggering downstream activation of the DAP12–SYK signaling cascade, which subsequently engages PI3K–AKT and MAPK pathways to initiate a neurogenic transcriptional program [76]. This reprogramming promotes the expression of neuronal markers such as TUBB3, NeuN, and other neurogenic factors, while concomitantly downregulating macrophage-specific genes. Importantly, genetic knockdown of TREM2 or pharmacologic blockade of its downstream kinases significantly mitigates MNT formation, reduces the integration of these cells into tumor-associated neural networks, and correlates with alleviation of chronic cancer-associated pain in preclinical models [77].

Functionally, MNT cells integrate into the tumor microenvironment not only as structural constituents of the newly formed neural networks but also as active signaling entities. These neuron-like cells can release neurotransmitters and neuropeptides, potentially forming aberrant neurocircuits within the tumor. Their dual macrophage-neuronal identity uniquely positions them to facilitate tumor innervation, reinforce tumor growth, and sustain pain signaling. Consequently, targeting key regulators of MNT cell formation, such as Smad3, TREM2, and POU4F1, provides a promising therapeutic strategy. Disrupting these pathways may effectively interfere with tumor–nerve interactions and relieve cancer-associated pain without impacting systemic nerve functions. Importantly, MNT has been validated not only in murine models using lineage tracing and single-cell RNA sequencing, but also in human tumor tissues. Clinical cohort analyses demonstrate the prognostic relevance of MNT across species. In lung cancer patients, increased abundance of MNT-like neuronally reprogrammed TAMs was associated with elevated cancer pain severity [12] and poor overall survival, while in nasopharyngeal carcinoma, APOE–TREM2–driven MNT differentiation correlated with chronic pain and unfavorable prognosis using both TCGA datasets and patient samples [77]. These findings support the presence and functional relevance of MNT in human cancers. Nonetheless, additional studies across diverse tumor types are warranted to further validate its clinical applicability. Consequently, targeting key regulators of MNT cell formation, such as Smad3, TREM2, and POU4F1, provides a promising therapeutic strategy. Disrupting these pathways may effectively interfere with tumor–nerve interactions and relieve cancer-associated pain without impacting systemic nerve functions.

### 2.3. Tumor–Nerve Interaction

Tumor-associated nerves are increasingly recognized as functional and dynamic components of the TME, playing active roles in cancer initiation, progression, and treatment resistance. These nerves—including sympathetic, parasympathetic, and sensory fibers—can infiltrate tumors via PNI or be newly generated through SNF. Their presence is not merely incidental; rather, these nerves establish bidirectional communication with cancer cells and other stromal elements [8,36,78], orchestrating critical processes such as tumor proliferation, angiogenesis, immune suppression, and metastasis [79,80].

Structurally, nerves serve as conduits for cancer cell dissemination. In PNI, tumor cells exploit pre-existing neural tracts to migrate into adjacent tissues or distant sites. Even prior to functional engagement, neural elements provide a scaffold for tumor spatial organization and microenvironmental compartmentalization [81,82]. Neurotrophic factors (e.g., NGF, BDNF, GDNF) and axon guidance molecules (e.g., semaphorins, netrins, ephrins) mediate this integration, promoting neural remodeling and facilitating tumor innervation [83,84]. Beyond structural roles, tumor-associated nerves shape the biochemical landscape of the TME through the secretion of neurotransmitters such as acetylcholine, NE, and neuropeptides (e.g., SP and CGRP). These neural signals activate oncogenic pathways (e.g., MAPK, PI3K–AKT), enhance tumor cell survival, and stimulate vascular and lymphatic remodeling [85]. Additionally, neural inputs suppress anti-tumor immunity by recruiting immunosuppressive myeloid cells or modulating T cell function, further enabling immune evasion [86]. Clinically, increased intratumoral nerve density is associated with more aggressive tumor phenotypes, higher recurrence rates, and poorer overall survival—particularly in pancreatic, prostate, breast, and head and neck cancers [25,47,87,88]. These observations underscore the prognostic significance of neural involvement and its potential utility as a biomarker for disease progression. While most clinical studies have quantified total nerve density using pan-neuronal markers such as S100B, NF-L, or PGP9.5 without stratifying by fiber subtype, preclinical data demonstrate that sympathetic, parasympathetic, and sensory nerves can exert distinct and sometimes opposing effects on tumor biology [87,89]. In colorectal cancer, subtype-specific denervation revealed that sympathetic fibers promote angiogenesis and immune suppression [90,91], parasympathetic fibers influence tumor cell proliferation through cholinergic pathways, and sensory fibers sustain tumor–nerve crosstalk and metastatic spread [92,93]. Similar findings have been reported in oral squamous cell carcinoma, where higher total nerve density predicted worse survival, surgical denervation suppressed tumor growth [87,94]. Supporting a mechanistic role for fiber subtype-specific influences, sympathetic denervation inhibited prostate cancer growth in in vivo orthotopic xenograft models through decreased norepinephrine signaling and reduced angiogenesis [95]. In gastric cancer, vagotomy or pharmacologic suppression of cholinergic signaling markedly attenuated tumor initiation and progression in Kras-driven mouse models, implicating parasympathetic nerves as key oncogenic drivers in gastric epithelium [96]. Likewise, sensory denervation using TRPV1-targeted approaches (e.g., resiniferatoxin) delayed PanIN-to-PDAC progression in genetically engineered mice by suppressing neuroepithelial crosstalk and downstream Stat3 signaling, underscoring the tumor-promoting role of sensory nerves in pancreatic cancer [97]. Collectively, these studies highlight the prognostic value of total nerve density in human cancers while providing a strong mechanistic rationale for future clinical investigations incorporating fiber-type-resolved nerve quantification.

Advances in technologies such as single-cell RNA sequencing, spatial transcriptomics, and tissue clearing have revealed unprecedented detail about tumor–nerve interactions, identifying neuron-like subpopulations and their spatial relationships with cancer and immune cells. This emerging field of cancer neurobiology opens new therapeutic possibilities: targeting neural inputs, neurotrophic signaling, or nerve–cancer crosstalk to attenuate disease progression and enhance treatment response. Understanding the multifaceted roles of tumor-associated nerves provides a conceptual framework for novel interventions aimed at disrupting cancer–nerve symbiosis—an area with immense translational potential. Importantly, the tumor-promoting effects of nerves are not limited to structural support or physical pathways; rather, they are largely mediated through the bioactive substances these nerves secrete. These neuronal secretions profoundly influence the tumor microenvironment, a topic explored in the following section.

## 3. Functional Roles of Neuronal Effectors in Cancer

Tumor-associated nerves are not passive structural elements but active modulators of cancer progression, influencing tumor growth, metastasis, immune evasion, and therapy resistance. One major mechanism by which nerves exert these effects is through the secretion of bioactive molecules, including neurotransmitters, neuropeptides, cytokines, and growth factors—that dynamically remodel the TME. These neuronal secretions act on a variety of cellular targets within the TME, such as cancer cells, immune cells, endothelial cells, and fibroblasts, thereby amplifying oncogenic signaling and reinforcing a pro-tumorigenic niche [88,98]. In this section, we highlight five key neuronal and neuroimmune mediators—NGF, BDNF, CGRP, norepinephrine (NE), and neuronal substance P (SP) that exemplify the diverse mechanisms through which nerve-derived signals shape the TME (Table 2).

### 3.1. NGF

NGF is a neurotrophic factor secreted by neurons, tumor cells, and stromal components, playing a critical role in promoting tumor innervation and progression. Through its high-affinity receptor, TrkA, NGF drives nerve sprouting and integration into the TME [99,100,101]. This process not only enhances neural density but also facilitates tumor–nerve crosstalk [123]. NGF activates key signaling pathways, including MAPK and PI3K/AKT, which promote cancer cell proliferation, survival, and migration [102,103,104,124]. Furthermore, NGF indirectly supports angiogenesis by upregulating vascular endothelial growth factor (VEGF), which contributes to tumor vascularization and sustains the growing tumor’s metabolic demands [105,125,126].

NGF’s impact on the TME extends to pain modulation, particularly in cancers like pancreatic and prostate cancer, where it is highly expressed [16,125,127,128,129]. Elevated NGF levels are associated with increased PNI, a hallmark of aggressive cancer behavior [130,131,132]. This neurotrophic signaling fosters a pro-tumorigenic environment by integrating nerves into the tumor stroma and facilitating metastatic dissemination through neural pathways [133,134,135].

Given its central role in tumor–nerve interactions, NGF is a promising therapeutic target. Preclinical studies have demonstrated the efficacy of NGF inhibitors and TrkA antagonists in reducing neural density, impairing tumor growth, and alleviating cancer-associated pain [136,137,138]. These findings highlight the therapeutic potential of disrupting NGF-TrkA signaling in highly innervated cancers.

### 3.2. BDNF

BDNF is a critical neurotrophic factor secreted by neurons and tumor cells that significantly influence tumor progression and TME. BDNF primarily exerts its effects through the TrkB receptor, which is expressed on cancer cells, nerves, and stromal components [104,106,107]. Activation of the BDNF-TrkB pathway enhances cancer cell proliferation, survival, and invasion by stimulating downstream signaling cascades, including PI3K/AKT [108,139], MAPK [109,140], and STAT3 pathways [110,141].

In the TME, BDNF promotes neural remodeling and integration, creating a supportive neurovascular niche [111,142]. It also facilitates tumor-associated neurogenesis, increasing neural density and fostering tumor–nerve crosstalk [143,144]. These interactions amplify oncogenic signaling and contribute to therapy resistance, as BDNF-TrkB signaling has been linked to reduced sensitivity to chemotherapy and targeted therapies [145,146,147]. Moreover, BDNF supports angiogenesis, further embedding neural and vascular components within the tumor to sustain its growth [148].

Elevated BDNF levels have been observed in aggressive cancers, such as glioblastoma [149], colorectal cancer [150], and non-small cell lung cancer (NSCLC) [150]. Clinically, high BDNF expression is associated with poor prognosis and advanced disease stages [151,152]. Targeting the BDNF-TrkB axis offers a promising therapeutic strategy, with preclinical models demonstrating that TrkB inhibitors reduce tumor growth, neural infiltration, and resistance to therapies [153,154,155]. These findings underscore the importance of BDNF in shaping the TME and its potential as a therapeutic target.

### 3.3. CGRP

CGRP is a neuropeptide primarily released by sensory neurons, playing a significant role in modulating the TME. CGRP has vasodilatory properties and directly influences tumor progression by promoting angiogenesis and vascular remodeling [126,156,157]. By enhancing blood flow and oxygen delivery within the TME, CGRP supports tumor growth and facilitates metastatic dissemination [158].

CGRP also plays a crucial role in immune modulation within the TME. It suppresses the activation of macrophages and T cells, impairing anti-tumor immune responses [112,113,114,159]. Simultaneously, CGRP fosters a tumor-promoting microenvironment by recruiting myeloid-derived suppressor cells (MDSCs) and inhibiting the dendritic cell (DC) development, which supports tumor survival and progression [159,160]. This immune evasion mechanism is particularly significant in cancers with high sensory nerve involvement, such as pancreatic, oral squamous cell carcinoma, head and neck cancers [115,161,162].

In addition to its immunosuppressive effects, CGRP enhances cancer cell migration and invasion, contributing to metastatic potential [163]. Elevated CGRP levels have been associated with increased recurrence and therapy resistance in certain cancers [164,165,166]. Targeting CGRP signaling presents a novel therapeutic approach, with potential strategies including CGRP antagonists or therapies aimed at sensory nerve modulation [113,159,167]. These interventions could disrupt the tumor-promoting effects of CGRP, offering new opportunities to improve outcomes in CGRP-associated cancers.

### 3.4. NE

NE, the primary catecholamine of the sympathetic nervous system (SNS), is a critical regulator of tumor–nerve interactions within the tumor microenvironment (TME) [116]. Released by sympathetic nerve fibers, NE binds to β2- and β3-adrenergic receptors expressed on tumor, stromal, and immune cells, activating cAMP–PKA signaling pathways that promote DNA repair, inhibit apoptosis, and enhance angiogenesis, immune evasion, and cancer progression [168,169]. Chronic stress and biobehavioral factors such as depression and low social support are associated with elevated intratumoral NE levels, which correlate with accelerated tumor growth and poor prognosis [117,170,171].

NE exposure induced BDNF expression, which acts on TrkB-expressing nerve fibers to drive tumor growth and establish a feedforward neurotrophic loop [172]. Similar mechanisms were observed in breast, pancreatic cancer and gastric cancers, where β-adrenergic signaling promoted VEGF and MMP expression and activated STAT3 and MAPK pathways, facilitating metastasis [118,133,173,174,175,176,177,178].

NE also upregulates NGF, particularly in pancreatic ductal adenocarcinoma, leading to enhanced neurite outgrowth and tumor innervation independently of β-adrenergic signaling [172]. Additionally, tumor-derived extracellular vesicles (TEX) from p53-deficient cancer cells lacking miR-34a-3p promote NE accumulation and dorsal root ganglion (DRG) neuron development and axon guidance [179].

Targeting the norepinephrine transporter (NET) has emerged as a promising theragnostic strategy [180]. The antidepressant drug venlafaxine (VEN) is used as an anticancer approach to improve the prognosis of colorectal cancer patients [181]. These findings underscore norepinephrine signaling as a central axis in tumor–nerve interactions.

### 3.5. SP

Substance P (SP) is a nociceptive neuropeptide released primarily by sensory nerves, where it exerts pro-inflammatory and pro-angiogenic effects in both physiological and pathological contexts [119,182]. SP mediates its actions through the neurokinin-1 receptor (NK-1R), a high-affinity receptor broadly expressed in both normal and neoplastic tissues [120]. Elevated expression of SP and NK-1R have been documented in various cancers, including leukemia, pancreatic, breast, and head and neck squamous cell carcinoma, where their signaling contributes to tumor proliferation, migration, and angiogenesis [183,184,185,186,187].

Within TME, SP promotes mast cell activation, vasodilation, and inflammation [122,188,189]. Notably, SP is not robustly produced by tumor cells themselves but is instead delivered via sensory nerve fibers [190]. In DRG coculture models, neurite-derived SP directly stimulates pancreatic cancer cell growth and motility [191]. NK-1R activation by SP also drives endothelial cell proliferation, promoting neovascularization in both in vitro and in vivo models [121].

Electrophysiological analyses of human tumors have revealed significantly elevated intratumoral electrical activity compared to benign or normal tissues, implying the presence of functional neuronal circuits. Tumors lacking nociceptor neurons in transgenic models exhibit diminished electrical activity and reduced growth, supporting a causal role for sensory nerves and SP in tumor progression. SP and NK-1R colocalization within tumor cells and vasculature further reinforces this mechanism [192]. The pharmacologic inhibition of NK-1R represents a promising therapeutic strategy to disrupt neurogenic support of malignancy and slow disease progression.

## 4. Therapeutic Strategies for Tumor Denervation

Neuronal secretions have been shown to actively promote cancer progression, suppress anti-tumor immunity, and contribute to resistance against conventional therapies [193]. These insights underscore the growing recognition of tumor–nerve interactions as a compelling therapeutic target in solid tumors. In response, a variety of strategies have been developed to disrupt both the molecular and structural elements of tumor innervation (Table 3). These approaches aim to inhibit nerve recruitment, block neurotrophic and axon guidance signaling, and neutralize the pro-tumorigenic effects mediated by neuronal activity within the tumor microenvironment.

### 4.1. Neurotrophic Signaling Blockade

Neurotrophic factors, including NGF and GDNF, are critical mediators of tumor innervation, playing a pivotal role in recruiting nerves and promoting neurogenesis within the TME. These factors interact with their respective receptors, such as TrkA (for NGF) and RET (for GDNF), to drive axonal growth and neural integration into tumors.

NGF-TrkA Inhibition: NGF inhibitors and TrkA antagonists represent a promising strategy to disrupt neurotrophic signaling. By blocking NGF-TrkA interactions, these therapies can reduce nerve infiltration into tumors and slow tumor growth [61,194]. Preclinical studies have demonstrated their efficacy in reducing neural density in aggressive cancers such as pancreatic [208] and prostate cancer [209], where high nerve infiltration correlates with poor prognosis. TrkA antagonists also mitigate nerve-related symptoms [210], such as cancer-associated pain, further enhancing their therapeutic value [211].

GDNF-RET Pathway Modulation: The GDNF-RET axis is another attractive target for neural inhibition. GDNF promotes nerve sprouting and survival, particularly in densely innervated cancers such as head and neck and colorectal cancers [195,212]. By targeting the RET receptor or its downstream signaling pathways, researchers have observed reduced nerve recruitment, decreased tumor proliferation, and limited metastatic potential in experimental models and clinical research [196,213,214,215,216]. Combining NGF-TrkA and GDNF-RET inhibitors may offer synergistic effects, addressing the redundancy often seen in neurotrophic signaling networks.

Blocking neurotrophic signaling disrupts the foundational mechanisms of tumor innervation, impairing both the recruitment of peripheral nerves and the formation of spontaneous nerve structures within tumors. However, neurotrophic factors are essential for normal sensory neuron survival and repair [217,218]. Systemic inhibition could cause sensory neuropathy, impaired wound healing, or autonomic dysfunction [219,220]. Tumor-restricted delivery and local administration strategies may mitigate these risks. Further research and clinical trials are necessary to validate the efficacy and safety of these inhibitors, with a focus on optimizing their use in combination with existing cancer therapies

### 4.2. Exosome Depletion

Tumor-derived exosomes, small extracellular vesicles secreted by cancer cells, play a significant role in tumor innervation. These exosomes are enriched with neurogenic factors, such as NGF, GDNF, and axon guidance molecules, which stimulate nerve recruitment and promote neurogenesis within the TME [197,221]. By acting as vehicles for intercellular communication, tumor-derived exosomes facilitate the integration of nerves into the tumor and amplify tumor–nerve interactions [222].

Inhibiting tumor exosome secretion—via Rab27A/B knockdown or GW4869 treatment—reduces nerve density and axonogenesis in tumors. Exosomes carrying EphrinB1 promote sensory nerve ingrowth, and targeting exosome biogenesis pathways impairs neural crosstalk, highlighting a therapeutic strategy to limit tumor innervation and progression [221,223].

Exosome-based therapies represent an innovative approach to disrupting tumor innervation by targeting the communication networks between cancer cells and nerves. Nevertheless, these exosomes also mediate physiological intercellular communication, including immune modulation and tissue repair [224,225,226]. Global exosome depletion could impair normal regeneration, immunity, and neuronal maintenance. Selective targeting of neurotrophic factor–enriched exosomes may reduce off-target effects [227]. Future research should focus on identifying biomarkers to predict exosome-mediated nerve recruitment and developing inhibitors that specifically target neurogenic exosomes without disrupting normal physiological processes.

### 4.3. Axon Modulation

Axon guidance molecules, including semaphorins, ephrins, and netrins, are essential regulators of neural development and integration. In the TME, these molecules are co-opted by tumors to promote nerve infiltration and SNF, driving cancer progression and metastasis [54,228,229]. Modulating these pathways offers a targeted approach to disrupt tumor–nerve interactions.

Semaphorins: Semaphorins, particularly class 3 semaphorins (SEMA3), act as repellent signals in axon guidance. Tumors downregulate these molecules or alter their signaling to promote neural infiltration [230]. Among class 3 semaphorins, SEMA3A, SEMA3F, and SEMA3C play distinct roles in tumor biology [231,232,233]. SEMA3A and SEMA3F function as tumor suppressors, inhibiting neural invasion, angiogenesis, and metastasis by antagonizing neuropilin/plexin signaling [232,233]. In contrast, SEMA3C is upregulated in cancers like prostate cancer and promotes tumor progression via EGFR, HER2, and MET activation through Plexin B1 [231,234]. Therapeutic strategies that restore SEMA3A/3F signaling or inhibit SEMA3C, such as semaphorin analogs, Plexin B1-Fc decoys, and anti-SEMA3C antibodies, have shown promise in reducing tumor growth and neural infiltration in preclinical cancer models [234,235].

Ephrins: Ephrin-Eph receptor signaling pathways are critical for neural integration within the TME [198]. In breast cancer, for instance, EphB4–ephrin-B2 forward signaling exerts tumor-suppressive effects, whereas ligand-independent EphB4 activity can promote tumor growth and invasion depending on the cellular context [199,221]. Dysregulated Eph expression is also implicated in drug resistance, especially in heterogeneous subtypes like triple-negative and HER2-positive breast cancers [236,237]. Targeting the Eph–ephrin axis with small-molecule inhibitors or ligand-blocking antibodies has shown promise in overcoming drug resistance and impairing tumor progression [238].

Netrins: Netrins are laminin-like axon guidance molecules that are frequently upregulated in cancers and contribute to neural invasion and tumor progression by interacting with receptors such as DCC, UNC5, and neogenin [200,239]. In gastric cancer, Netrin-1 expression correlates with neural invasion and lymph node metastasis, and silencing Netrin-1 or its receptor neogenin significantly impairs cancer cell migration along neurites both in vitro and in vivo [240,241]. In endometrial cancer models, pharmacologic blockade of Netrin-1 using the monoclonal antibody NP137 induces apoptosis, suppresses EMT, and reduces tumor burden in both preclinical and early-phase clinical settings [242]. These findings underscore the therapeutic potential of targeting Netrin-1 signaling to limit tumor–nerve crosstalk and invasive behavior [240,242].

By modulating axon guidance molecules, these strategies directly interfere with the mechanisms that enable neural infiltration and SNF. However, semaphorin, ephrin, and netrin signaling are critical for normal nervous system maintenance and regeneration, and systemic modulation could disrupt neural homeostasis and developmental pathways [243,244]. Localized delivery or transient modulation may therefore be preferable to minimize systemic neurotoxicity. Continued research is needed to optimize these therapies and balance therapeutic efficacy with safety.

### 4.4. Targeting TRPV1

Transient receptor potential vanilloid 1 (TRPV1) is a non-selective cation channel highly expressed in nociceptive sensory neurons, where it mediates the detection of noxious heat and inflammatory signals [245]. In tumor contexts, TRPV1-expressing sensory fibers contribute to cancer-associated pain and may promote tumor progression through neuropeptide release, such as substance P and CGRP. Pharmacological TRPV1 antagonists and genetic ablation approaches have demonstrated significant analgesic and anti-tumor effects in preclinical cancer models [85,201].

However, TRPV1 is also expressed in non-neuronal cells, including immune cells, vascular endothelium, and epithelial tissues, where it participates in thermoregulation, vasodilation, and immune modulation. Systemic TRPV1 blockade in clinical trials has been associated with hyperthermia and impaired noxious heat perception, raising the risk of accidental thermal injury [246,247]. Therefore, future therapeutic strategies should aim to restrict TRPV1 inhibition to the tumor microenvironment—for example, by using nanoparticle-mediated delivery, ligand-conjugated inhibitors, or localized ablation—to maximize efficacy while minimizing systemic adverse effects.

Given that TRPV1 is predominantly expressed in sensory neurons, indiscriminate inhibition may result in off-target effects on peripheral sensory fibers not involved in tumor biology [248,249]. Recent studies suggest that TRPV1 expression patterns may vary across tumor types and anatomical sites, further underscoring the need for precise, context-specific targeting [250,251]. Future development of sensory nerve subtype-specific imaging or transcriptomic profiling could guide more accurate TRPV1-targeted interventions and minimize unintended neurotoxicity [252].

### 4.5. Targeting Adrenergic Signaling

Adrenergic nerves within the tumor microenvironment release norepinephrine (NE), which activates β2- and β3-adrenergic receptors on tumor cells and stromal components, promoting angiogenesis, immunosuppression, and metastatic spread. β-adrenergic antagonists, such as propranolol, have shown anti-tumor activity in preclinical models and retrospective clinical studies, with potential benefits including reduced angiogenesis and enhanced anti-tumor immunity [202,203,204,253].

Nonetheless, systemic β-blockade carries inherent risks, such as bradycardia, hypotension, bronchospasm, and interference with physiological stress responses, which may limit its use in certain patient populations [254]. Moreover, prolonged adrenergic blockade can induce compensatory neuroplasticity, including increased sympathetic sprouting or recruitment of alternative neurotransmitter pathways, potentially diminishing long-term efficacy [255]. Precision targeting approaches—such as local injection, tumor-specific ligand conjugation, or image-guided nerve ablation—are needed to harness the therapeutic benefits of adrenergic inhibition while minimizing systemic toxicity.

Importantly, β2- and β3-adrenergic receptors are not uniformly distributed across all nerve subtypes or tumor compartments. Without spatial and molecular resolution, systemic β-blockade risks interfering with beneficial neural circuits or triggering compensatory responses. Therefore, incorporation of nerve subtype–selective strategies, such as imaging-based fiber mapping or ligand-targeted delivery guided by adrenergic receptor localization, will be crucial for maximizing therapeutic benefit while avoiding collateral damage.

### 4.6. Direct Denervation

Denervation techniques involve the surgical or chemical depletion of nerves within tumors or their surrounding microenvironment to disrupt tumor–nerve interactions. These strategies aim to reduce neural density, impair tumor progression, and alleviate nerve-associated symptoms such as cancer-related pain.

Surgical Denervation: Surgical transection of sympathetic or parasympathetic nerves has been explored in preclinical cancer models [96]. For instance, in breast and gastric cancers, nerve transection significantly reduced tumor growth and metastasis [42]. Surgical denervation may also enhance the efficacy of other therapies by disrupting neural support for cancer cells.

Chemical Denervation: Chemical agents, such as 6-hydroxydopamine (6OHDA), selectively ablate sympathetic nerves, effectively reducing neural input to the tumor [205]. Neonatal capsaicin slows development of PNI and prolongs survival time [256]. From a therapeutic perspective, targeting key molecular drivers of spontaneous neurogenesis—including NGF, GDNF, VEGF, and the TGF-β/Smad3 pathway underlying MNT—represents a promising approach to mitigating the adverse effects of tumor innervation. Inhibiting these pathways may disrupt intratumoral nerve networks, impair cancer progression, and alleviate associated cancer-related pain, thereby enhancing patient outcomes [257]. Preclinical studies in prostate and breast cancer models have demonstrated that chemical denervation not only impairs tumor progression but also enhances the anti-tumor immune response by reducing immunosuppressive signaling mediated by nerves [258]. Importantly, Surgical or chemical denervation may induce compensatory neuroplasticity, collateral sprouting, or functional deficits in surrounding normal tissues. Side effects such as autonomic dysregulation, sensory loss, or chronic pain syndromes should be considered [259]. Image-guided, tumor-specific approaches could reduce systemic complications [260].

Denervation approaches offer a direct and effective means of targeting tumor-associated nerves. However, these strategies face challenges, including potential compensatory mechanisms through alternative neural pathways and the risk of unintended side effects on normal physiological functions. Combining denervation with therapies targeting neurotrophic factors or axon guidance molecules could provide a synergistic effect, ensuring comprehensive disruption of tumor–nerve interactions while minimizing adverse effects [261]. Further clinical trials are needed to validate these approaches and determine their long-term safety and efficacy. Overall, the clinical translation of denervation-based strategies will require careful balancing of efficacy with safety, addressing risks such as sensory deficits, autonomic dysfunction, pain exacerbation, and delivery challenges, before integration into routine oncology practice.

### 4.7. Fiber-Specific Denervation and Imaging-Guided Nerve Mapping

The heterogeneity of tumor-infiltrating nerves—comprising sympathetic, parasympathetic, and sensory subtypes—necessitates strategies that can selectively target pathogenic fibers while preserving physiological neural circuits. Traditional denervation and pharmacologic blockade approaches often lack such specificity, potentially leading to systemic toxicity or compensatory neural remodeling.

Fiber-specific ablation techniques, such as optogenetic or chemogenetic tools, have shown promise in preclinical neuroscience for modulating distinct nerve types [206,252,262]. Though not yet widely translated to oncology, these technologies could be adapted to selectively ablate tumor-supportive nerves (e.g., β-adrenergic or TRPV1-positive sensory fibers) based on their molecular or anatomical signatures.

Imaging-based nerve mapping represents a complementary strategy to guide intervention. Advanced modalities such as high-resolution MRI, PET tracers targeting neuronal markers (e.g., radiolabeled NET ligands), and intraoperative nerve-labeling dyes can help visualize nerve distribution within tumors [207,263,264]. These approaches not only facilitate selective denervation but also enable real-time monitoring of nerve density changes during treatment. Integrating fiber-specific and imaging-guided techniques will be critical for minimizing off-target effects, avoiding collateral damage to adjacent healthy nerves, and maximizing the therapeutic window for tumor denervation strategies.

## 5. Conclusions

Tumor innervation is increasingly recognized as a critical, though historically underappreciated, driver of cancer progression, metastasis, immune evasion, and cancer-associated pain. Far from being passive structural elements, nerves actively participate in the tumor microenvironment through dynamic crosstalk with cancer cells and stromal components. This review highlights a key conceptual advance by delineating two distinct modes of tumor innervation: PNI, characterized by cancer cells migrating along pre-existing nerves, and SNF, wherein nerves are generated de novo within tumors through processes such as neurogenesis and axonogenesis. This mechanistic distinction clarifies the complexity of neural contributions to cancer biology and lays the foundation for more targeted, pathway-specific interventions.

Neuronal secretions—including NGF, BDNF, CGRP, norepinephrine, and neuronal substance P—serve as potent mediators of tumor–nerve interactions. These factors not only recruit and stimulate neural elements but also modulate key components of the tumor microenvironment, including immune suppression, angiogenesis, and metastasis. Their context-specific effects vary by tumor type and innervation pattern, underscoring the need for precision approaches to therapeutic targeting.

Among the most striking discoveries in this field is the phenomenon of MNT, wherein TAMs adopt neuronal phenotypes under the influence of TGF-β/Smad3 or APOE–TREM2 signaling. These MNT cells express neuronal markers, integrate into intratumoral neural networks, and secrete neurotransmitters, functioning as both structural and functional surrogates of neurons. Beyond their contribution to tumor innervation, MNT cells have been directly implicated in the generation of cancer-associated nociceptive pain. Unlike previously described indirect effects of macrophages on pain through inflammatory mediators, MNT cells exhibit active neuronal properties—including calcium signaling, TRPV1/TRPV4-mediated nociceptive activity, and synaptotagmin-driven neurotransmitter release—that establish aberrant neurocircuits within the TME. Pharmacologic and macrophage-specific inhibition of Smad3 not only blocks MNT formation but also markedly alleviates spontaneous nocifensive behavior in tumor-bearing mice, suggesting that targeting MNT may serve as a dual-function strategy to suppress both tumor progression and cancer pain. The identification of MNT not only expands our understanding of myeloid plasticity but also opens a promising therapeutic avenue by disrupting this neuroimmune axis [12].

Despite these advances, major challenges remain. The temporal dynamics of tumor neurogenesis, tissue-specific neural responses, and the identification of tumor-specific neural circuits all require further elucidation. Moreover, the development of selective strategies that target intratumoral nerves without impairing systemic neural function remains a significant hurdle. Emerging tools such as spatial transcriptomics, optogenetics, and electroceutical modulation hold promise for precise, minimally invasive manipulation of tumor-associated nerves.

In addition to these scientific and technical challenges, clinical translation demands well-defined patient selection criteria to stratify individuals most likely to benefit from innervation-targeted therapies [265]. Potential strategies include tissue-based assessment of intratumoral nerve density using pan-neuronal markers such as PGP9.5 and TUBB3, which correlate with poor prognosis across multiple cancer types [89,266]. Molecular profiling of tumor tissue or exosomes for neurotrophic factors (NGF, BDNF, GDNF), chemokines (CXCL12, CCL2), or neuronal transcription factors (POU4F1) may serve as predictive biomarkers of neural involvement. Circulating neuropeptides, including substance P and CGRP, could provide minimally invasive blood-based indicators of tumor–nerve interactions [115]. In addition, advanced imaging modalities such as PET or MRI with nerve-specific tracers, and functional neuroinflammation imaging [267], may help visualize intratumoral neural networks in vivo. Integrating these biomarkers into clinical workflows will facilitate patient stratification, optimize trial design, and support the rational implementation of nerve-targeted interventions. These clinical considerations underscore the translational relevance of tumor–nerve biology and set the stage for future clinical application.

In conclusion, tumor innervation—through both PNI and SNF—is increasingly recognized as a critical component of cancer biology. It intersects with immune, vascular, and stromal networks, and contributes directly to disease progression and symptom burden. A deeper understanding of tumor–nerve interactions, including mechanisms such as MNT, will be essential to unlocking novel, mechanism-based strategies for cancer therapy. Ultimately, targeting the neural infrastructure of malignancy may offer transformative clinical benefits—not only in halting cancer growth but also in addressing one of its most devastating complications: chronic cancer pain.

## Figures and Tables

**Table 1:** Tumor Types and Innervation Characteristics.

Cancer Type	Innervation Characteristics	Key Findings and Implications	Reference
Pancreatic Cancer	Nearly 100% of cases exhibit dense nerve infiltration and PNI.	Strong correlation with poor prognosis, recurrence, and pain; neural crosstalk promotes angiogenesis and metastasis.	[17,32,33,34]
Prostate Cancer	High nerve density, with sympathetic and parasympathetic involvement.	Sympathetic nerves promote growth; parasympathetic nerves promote metastasis; PNI is a survival risk factor.	[18,19,35]
Colorectal Cancer	~33% show PNI and tumor-associated nerve infiltration.	High nerve density linked to recurrence, poor survival; nerves serve as dissemination routes.	[25,30,36,37]
Head and Neck Cancer	Up to 80% exhibit PNI, especially in aggressive subtypes.	Associated with local recurrence, reduced survival, immune suppression, and progression.	[24,38]
Gastric Cancer	Exhibits both peripheral nerve infiltration and spontaneous nerve formation.	High nerve density linked to metastasis and poor prognosis; NGF-mediated innervation may drive malignancy.	[39]
Cervical Cancer	PNI observed in subsets, often in large or late-stage tumors.	Neural infiltration enhances tumor aggressiveness and worsens clinical outcomes.	[40]
Glioblastoma	Tumor cells directly induce spontaneous neurogenesis.	Glioma-neuron networks promote proliferation, angiogenesis, and treatment resistance.	[6,8,20,21]
Breast Cancer	Less common neural infiltration, but observed in aggressive subtypes.	Sympathetic signaling drives tumor growth; axon guidance molecules contribute to metastasis.	[41,42]
Melanoma	Neural association linked to neural crest origin.	PNI enhances invasion depth and therapy resistance.	[10]
NSCLC	Tumors co-opt neural elements via metastatic niche formation (MNT).	Tumor-associated nerves promote immune suppression and angiogenesis.	[3,43]

**Table 2:** Functional roles of Neuronal Effectors in Cancer.

Neuronal Factor	Receptor(s)	Function in TME	Key Tumor-Associated Effects	Reference
NGF	TrkA	Promotes tumor innervation, neurogenesis, angiogenesis	Enhances PNI, tumor growth, metastasis, and pain	[99,100,101,102,103,104,105]
BDNF	TrkB	Stimulates PI3K/AKT, MAPK, STAT3 pathways	Promotes tumor proliferation, therapy resistance, angiogenesis	[106,107,108,109,110,111]
CGRP	RAMP1/CLR complex	Induces vasodilation, suppresses immune cells	Facilitates angiogenesis, immune evasion, metastasis, therapy resistance, pain	[112,113,114,115]
NE	β2/β3-AR (Adrenergic Receptors)	Activates cAMP–PKA, induces BDNF and NGF	Enhances DNA repair, immune evasion, angiogenesis, metastasis, pain	[116,117,118]
SP	NK-1R	Promotes inflammation, angiogenesis, nerve activity	Enhances proliferation, motility, vascularization, tumor pain	[119,120,121,122]

**Table 3:** Neuro-Targeted Therapeutic Strategies in Tumors.

Strategy	Mechanism	Examples	Potential Outcomes	Clinical Status	Reference
Blocking Neurotrophic Signaling	Inhibits nerve growth and recruitment by targeting neurotrophic factors and their receptors.	1. NGF-TrkA inhibitors (e.g., NGF monoclonal antibodies, TrkA antagonists). 2. GDNF-RET inhibitors (e.g., RET kinase inhibitors).	Reduces nerve density, impairs tumor growth, and alleviates cancer-associated pain.	TrkA: Phase II; RET inhibitors: approved for RET + cancers	[61,194,195,196]
Exosome-Based Therapies	Disrupts tumor-derived exosome production, release, or uptake to block neurogenic signaling.	1. Inhibitors of Rab GTPases (e.g., Rab27a blockers). 2. Targeting exosome receptors on nerves.	Reduces nerve recruitment and spontaneous nerve formation within tumors.	Preclinical	[196,197]
Axon Guidance Molecule Modulation	Inhibits pathways involved in nerve growth and integration into tumors.	1. Semaphorin-based therapies (e.g., semaphorin analogs). 2. Ephrin inhibitors (e.g., Eph receptor antagonists). 3. Netrin-targeting agents (e.g., netrin receptor blockers).	Prevents neural infiltration, reduces tumor-supportive nerve networks, and limits metastasis.	Preclinical	[198,199,200]
Targeting TRPV1	Blocks nociceptive sensory neuron signaling and neuropeptide release by inhibiting TRPV1 channel activity.	TRPV1 antagonists, TRPV1 gene ablation approaches.	Reduces cancer-associated pain and may impair tumor-supportive neural activity; risks include hyperthermia and impaired heat sensation.	Preclinical/early clinical trials	[85,201]
Targeting Adrenergic Signaling	Blocks norepinephrine-mediated β2/β3-adrenergic receptor signaling to reduce tumor-promoting neural effects.	β-blockers (e.g., propranolol), adrenergic nerve ablation.	Reduces angiogenesis, immunosuppression, and metastasis; risks include cardiovascular side effects and compensatory sympathetic sprouting.	Propranolol: repurposed in clinical studies; others: preclinical	[202,203,204]
Denervation Approaches	Ablates nerves physically or chemically to disrupt tumor–nerve interactions.	1. Surgical denervation (e.g., nerve transection in pancreatic cancer). 2. Chemical denervation (e.g., 6-hydroxydopamine for sympathetic nerve ablation).	Reduces tumor progression, enhances therapy response, and alleviates neural contributions to tumor-supportive environments.	Preclinical/limited clinical experience	[200,205]
Fiber-Specific Denervation and Imaging-Guided Mapping	Targets specific nerve subtypes based on molecular markers; enables visualization of intratumoral nerve architecture	TRPV1+/β-AR+ fiber ablation, PET imaging, optogenetics	Enhances selectivity, reduces collateral damage, enables precision denervation	/	[206,207]

## Abbreviations

The following abbreviations are used in this manuscript:

AKT	Protein kinase B
ANO1	Anoctamin-1 (calcium-activated chloride channel)
APOE	Apolipoprotein E
BDNF	Brain-derived neurotrophic factor
CAFs	Cancer-associated fibroblasts
cAMP	Cyclic adenosine monophosphate
CGRP	Calcitonin gene-related peptide
DCX	Doublecortin
DCC	Deleted in colorectal carcinoma
GDNF	Glial cell line-derived neurotrophic factor
GPCR	G protein–coupled receptor
ICD	Intracellular domain
MAP1B	Microtubule-associated protein 1B
MAPK	Mitogen-activated protein kinase
MMP	Matrix metalloproteinase
MNT	Macrophage to neuron-like cell transition
NE	Norepinephrine
NeuN	Neuronal nuclei
NGF	Nerve growth factor
NK1R	Neurokinin-1 receptor
p75NTR	p75 neurotrophin receptor
PI3K	Phosphoinositide 3-kinase
PKA	Protein kinase A
PNI	Perineural invasion
POU4F1	POU domain, class 4, transcription factor 1 (BRN3A)
RET	Rearranged during transfection (RET receptor tyrosine kinase)
RAMP1	Receptor activity-modifying protein 1
SHANK	SH3 and multiple ankyrin repeat domains protein
Smad3	Mothers against decapentaplegic homolog 3
SNF	Spontaneous nerve formation
SP	Substance P
STAT3	Signal transducer and activator of transcription 3
TAMs	Tumor-associated macrophages
TGF-β1	Transforming growth factor beta 1
TRPV1	Transient receptor potential vanilloid 1
TRPV4	Transient receptor potential vanilloid 4
TrkA	Tropomyosin receptor kinase A
TrkB	Tropomyosin receptor kinase B
TUBB3	β3-tubulin
VEGF	Vascular endothelial growth factor
